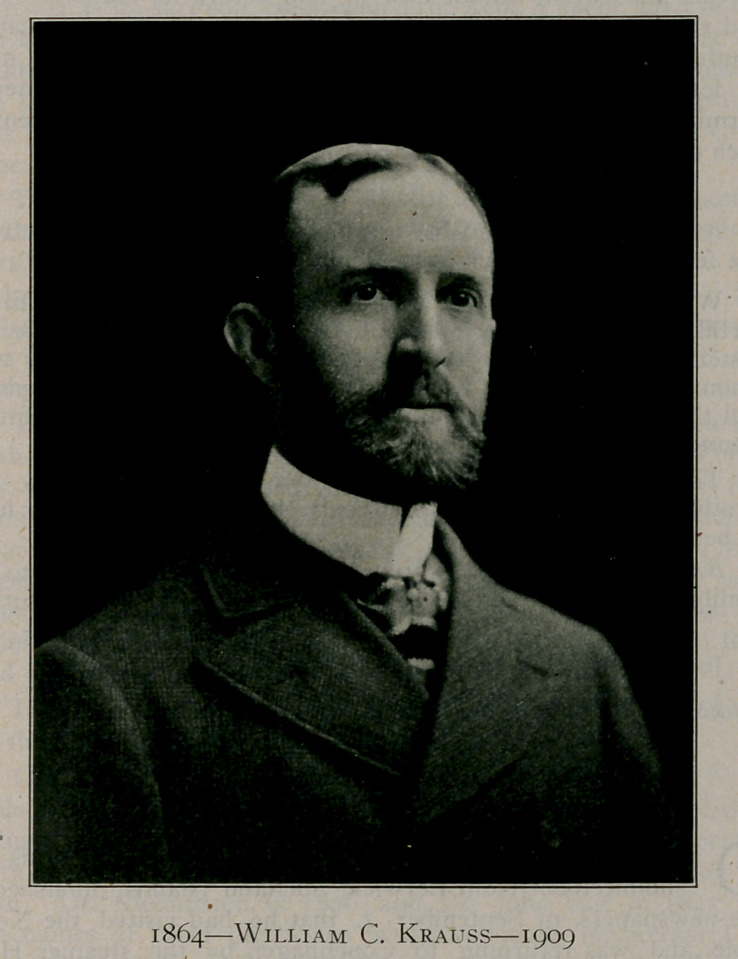# Dr. William C. Krauss

**Published:** 1909-10

**Authors:** 


### BUFFALO MEDICAL JOURNAL.

**A Monthly Review of Medicine and Surgery.**

*EDITOR*

WILLIAM WARREN POTTER, M. D.

**All communications, whether of a literary or business nature, books for review and exchanges, should be addressed to the editor, 238 Delaware Avenue, Buffalo, N.Y.**

Vol. Lxv. OCTOBER, 1909. No. 3

### Dr. William C. Krauss

AS this issue, of The Buffalo Medical Journal goes to press word is received of the sudden death in New York City on September 21, 1909, of William C. Krauss, M. D., an assistant editor of the Journal, and one of the most prominent physicians in western New York. Details of the last illness of Dr. Krauss are lacking. All that is known is that he died at a New York Hospital, to which he was taken from the steamer on which he returned from Europe on September 20, and that death was due to cardiac disease with which he was afflicted and for relief of which he went abroad last July.

Letters recently received from Dr. Krauss indicated little or no improvement in his condition. The meager information at hand is to the effect that his condition became alarming as the steamer neared New York, and a wireless message was sent to port summoning a physician to meet him. On the arrival of the vessel Dr. Krauss was immediately removed to the hospital where he died a few hours later, Mrs. Krauss having been summoned from Buffalo when it was found that her husband's condition was grave. Dr. Krauss is survived by his wife and three children.

Dr. Krauss was born at Attica, N. Y., in 1864. He attended the public schools there and later entered Cornell, from which he was graduated in 1884. In 1886 he received the degree of M. D., at Bellevue. He spent several years in Germany, and on the completion of his studies in 1889 was granted a degree.

He came to Buffalo and established himself in practice confining his work to diseases of the nervous system, in which branch of medicine he achieved an enviable reputation and became a recognised authority. He was president of the board of trustees of the Buffalo State Hospital for the insane, chief physician at the Providence Retreat, neurologist to the Buffalo General Hospital, the Erie County Hospital, the German Hospital and the

Emergency Hospital. He was a member of the Buffalo University and Pioneers' Club, the Buffalo Academy of Medicine, the Medical Union, and Washington Lodge, F. and A. M., and was a Scottish Rite Mason.

To the literature of medicine, more particularly that dealing with mental and nervous diseases, Dr. Krauss contributed many

1864—William C. Krauss—1909

valuable papers. Prior to his departure for Europe, he completed the writing of a book on the causes and treatment of insanity, which was to have been delivered to his publishers on his return. He also had in course of preparation, and nearing completion, an extensive and exhaustive study of spinal cord tumors, which it was his intention to publish early in the coming year. His contributions to the Buffalo Medical Journal, consisting mainly of editorials and reviews, were marked by a scholarly finish and a literary grace seldom developed in one whose life has been devoted wholly to scientific pursuits. His style was simple, clear and convincing; his logic was unassailable.

The JOURNAL regrets that there cannot be written at this time a more fitting tribute to the life and works of Dr. Krauss; that its full appreciation of his invaluable assistance in the conduct of this publication cannot now be laid before its readers. That and those tributes which are the dues of a courtly gentleman, a gentle physician and a warm-hearted friend and associate, must be left for another time. For the present, opportunity merely permits of simple announcement, and a fragmentary statement of such chronologic facts as may be hastily gathered.

---

At a meeting of the Board of Trustees of Providence Retreat, the following resolutions were adopted:

WHEREAS, God in His infinite wisdom has removed from his earthly labors our superintendent, Dr. William C. Krauss, we, the Sisters in charge of Providence Retreat, also the physicians with whom the late Doctor Krauss was associated for several years, feel the loss most keenly, and desire to record the fact in befitting manner.

Therefore, *Be it Resolved,* That we tender the widow and family of the deceased our most heartfelt sorrow in this their hour of bereavement.

*Be it Resolved,* That a copy of these resolutions be sent to the family of deceased, and also a copy kept on file at the institution.

SISTERS OF CHARITY.

JOHN J. TWOHEY, Physician-in-Charge.

JAMES I. KEARNEY, Resident Physician.

## Figures and Tables

**Figure f1:**